# The impact of nurse-driven targeted HIV screening in 8 emergency departments: study protocol for the DICI-VIH cluster-randomized two-period crossover trial

**DOI:** 10.1186/s12879-016-1377-6

**Published:** 2016-02-01

**Authors:** Judith Leblanc, Alexandra Rousseau, Gilles Hejblum, Isabelle Durand-Zaleski, Pierre de Truchis, France Lert, Dominique Costagliola, Tabassome Simon, Anne-Claude Crémieux

**Affiliations:** 1 Assistance Publique – Hôpitaux de Paris (AP-HP), Groupe Hospitalier des Hôpitaux Universitaires Est Parisien, Clinical Research Center of East of Paris (CRC-Est), F75012 Paris, France; 2 Université Paris Saclay - Université Versailles Saint-Quentin, Doctoral School of Public Health (EDSP), UMR 1173, F92380 Garches, France; 3 AP-HP, Groupe Hospitalier des Hôpitaux Universitaires Est Parisien, Clinical Research Unit of East of Paris (URC-Est), F75012 Paris, France; 4 Sorbonne Universités, UPMC Univ Paris 06, INSERM, Institut Pierre Louis d’Épidémiologie et de Santé Publique (IPLESP UMRS 1136), F75012 Paris, France; 5 AP-HP, Hôpital Hôtel-Dieu, URC Eco Île-de-France, F75004 Paris, France; 6 Université Paris Diderot, Univ Paris 07, INSERM, ECEVE, UMR 1123, F75019 Paris, France; 7 AP-HP, Hôpital Henri-Mondor, Santé publique, F94010 Créteil, France; 8 AP-HP, Hôpital Raymond-Poincaré, Infectious Disease Department, F92380 Garches, France; 9 Université Paris Sud, Univ Paris 11, INSERM, Centre for research in Epidemiology and population health, U 1018, F94800 Villejuif, France; 10 AP-HP, Groupe Hospitalier des Hôpitaux Universitaires Est Parisien, Department of clinical pharmacology and Clinical Research Center of East of Paris (CRC-Est), F75012 Paris, France; 11 Sorbonne Universités, UPMC Univ Paris 06, INSERM, UMR 1148, F75018 Paris, France; 12 Université Versailles Saint-Quentin, UMR 1173, F92380 Garches, France

**Keywords:** Decision support techniques, Emergency service, Hospital, HIV, HIV infections, Mass screening, Nurses, Nursing, Prevention and control, Randomized controlled trial, Risk factors

## Abstract

**Background:**

In 2010, to reduce late HIV diagnosis, the French national health agency endorsed non-targeted HIV screening in health care settings. Despite these recommendations, non-targeted screening has not been implemented and only physician-directed diagnostic testing is currently performed. A survey conducted in 2010 in 29 French Emergency Departments (EDs) showed that non-targeted nurse-driven screening was feasible though only a few new HIV diagnoses were identified, predominantly among high-risk groups. A strategy targeting high-risk groups combined with current practice could be shown to be feasible, more efficient and cost-effective than current practice alone.

**Methods/Design:**

DICI-VIH (acronym for nurse-driven targeted HIV screening) is a multicentre, cluster-randomized, two-period crossover trial. The primary objective is to compare the effectiveness of 2 strategies for diagnosing HIV among adult patients visiting EDs: nurse-driven targeted HIV screening combined with current practice (physician-directed diagnostic testing) versus current practice alone. Main secondary objectives are to compare access to specialist consultation and how early HIV diagnosis occurs in the course of the disease between the 2 groups, and to evaluate the implementation, acceptability and cost-effectiveness of nurse-driven targeted screening. The 2 strategies take place during 2 randomly assigned periods in 8 EDs of metropolitan Paris, where 42 % of France’s new HIV patients are diagnosed every year. All patients aged 18 to 64, not presenting secondary to HIV exposure are included. During the intervention period, patients are invited to fill a 7-item questionnaire (country of birth, sexual partners and injection drug use) in order to select individuals who are offered a rapid test. If the rapid test is reactive, a follow-up visit with an infectious disease specialist is scheduled within 72 h. Assuming an 80 % statistical power and a 5 % type 1 error, with 1.04 and 3.38 new diagnoses per 10,000 patients in the control and targeted groups respectively, a sample size of 140,000 patients was estimated corresponding to 8,750 patients per ED and per period. Inclusions started in June 2014. Results are expected by mid-2016.

**Discussion:**

The DICI-VIH study is the first large randomized controlled trial designed to assess nurse-driven targeted HIV screening. This study can provide valuable information on HIV screening in health care settings.

**Trial registration:**

ClinicalTrials.gov: NCT02127424 (29 April 2014).

## Background

Optimizing HIV screening and reducing late diagnosis remain challenging issues in most countries [1–4]. Indeed, late HIV diagnosis is associated with increased mortality [5]. Moreover, the early introduction of antiretroviral treatment provides net benefits on both morbidity and mortality and reduces secondary transmission [6–8].

In France, although 5 million HIV screening tests are performed each year [9], 25 % of new HIV diagnoses were discovered at a late stage in 2013 (<200 CD4/mm^3^ or AIDS stage) [1]. Until 2010, screening strategies aimed to encourage voluntary testing in specialist clinics or through a primary care physician. In 2010, mirroring what had already been suggested in other countries [10, 11], national recommendations encouraged French healthcare staff to conduct non-targeted screening among the 15 to 70 year old population, in order to reach patients who wouldn’t voluntarily get tested [12, 13]. In this context, nurses were given the possibility of offering, performing and delivering the result of an HIV rapid test [14].

A study conducted in the metropolitan Paris area evaluated the effectiveness of nurse-driven non-targeted HIV screening in 29 public hospital Emergency Departments (EDs) [15]. EDs were considered to be an ideal setting to evaluate HIV screening strategies as they receive an average of 18.5 million visits annually, representing over 25 % of the general population, including low-income groups, the uninsured and other minorities at highest risk of HIV exposure [16]. This study concluded that non-targeted HIV screening was feasible and well accepted by patients [15, 17]. However, only 18 patients out of 12,754 tested patients were newly diagnosed, yielding a prevalence of 0.14 % (95 % CI [0.08 %–0.22 %]). These patients belonged to the highest risk groups (i. e. men who have sex with men (MSM), immigrants from generalized epidemic areas). These results and those from other studies conducted in France, in the United States and the United Kingdom showed that non-targeted screening was feasible in health care settings and that it provided a means to reach a large number of patients [18–20]. Nevertheless, the corresponding effectiveness in diagnosing HIV is subject to debate [15, 18, 21–23].

Studies evaluating nurse-driven HIV screening, compared to screening performed by other health care staff, showed a trend in higher test offering, better acceptance and higher delivery rates with the implementation of nurse-driven HIV screening [24]. However, a progressive decrease in test offering rates from nurses and other staff over time has been observed, most likely resulting from of a loss of motivation related to the small number of new diagnoses, which were concentrated in patients at high risk of HIV exposure [20, 25, 26]. It has been suggested that nurse-driven HIV screening in EDs was possible on a wider scale over the long-term but would be more feasible if it targeted a specific patient population [27].

Recent studies conducted in the United States, the United Kingdom and Spain have supported targeted screening efforts [28–32]. Indeed, in these countries, the hidden epidemic is less prevalent than expected [33] and is, overall, concentrated in the highest risk groups [15, 18, 34, 35]. The studies showed that targeted screening was associated with identification of newly HIV diagnoses when compared to non-targeted screening [28, 31]. Only one study, conducted in a single centre, did not observe any benefit from using a targeted strategy [36]. However, in these studies, less than 50 % of the population identified as being at high risk of HIV exposure were actually tested [31, 36] and cost-effectiveness analysis was not always provided, thus requiring prospective multicentre trials with cost-effectiveness analysis.

### Rationale for the study design

The DICI-VIH study (a French acronym for *Dépistage infirmier ciblé du VIH, n*urse-driven targeted HIV screening) is a multicentre, cluster-randomized two-period crossover trial comparing the effectiveness of targeted screening combined with current practice to that of current practice alone. Several study designs can be explored to evaluate the effectiveness of targeted screening in health care settings. It could be compared either to non-targeted screening or to current practice, which is most often limited to diagnostic testing. However, given both the low frequency of new diagnoses in non-targeted screening strategies and the burden of its implementation, non-targeted screening is not likely to be carried out in France. Indeed, the results of a questionnaire submitted in February 2013 showed that none of the EDs included in the present study had adopted it. Moreover, studies show that non-targeted screening has not been implemented in the United States or in the United Kingdom several years after the publication of the recommendations [26, 31, 37, 38]. It therefore seems relevant to evaluate the effectiveness of a combined strategy of targeted screening and current practice in comparison with current practice alone in order to draw conclusions on whether or not it is worthwhile to recommend this strategy in EDs.

Individual randomization in each ED would require the intermittent involvement of nurses in targeted screening, raising practical and organizational concerns, as well as generalizability issues since a triage nurse would never be able to intermittently apply the targeted strategy. Therefore, a cluster-randomized design was chosen to compare the 2 strategies. The centres were selected based on the overall high risk of exposure to HIV of their patient populations (see Study settings) and were assumed to differ in terms of organization and care practices. Thus, a cluster-randomized two-period crossover design, in which all clusters apply each strategy over 2 distinct periods (the objective of the randomization process being to randomly assign which strategy is applied first in each cluster), enables the observation of matched-pair differences between the strategies within each ED [39, 40].

### Primary objective

To compare the effectiveness of 2 strategies for diagnosing HIV among adult patients visiting EDs: nurse-driven targeted HIV screening combined with current practice (physician-directed HIV diagnostic testing) versus current practice alone.

### Secondary objectives

The secondary objectives are to compare the 2 groups in terms of:

- Access to a specialist consultation within 3 months following the HIV diagnosis,
- Proportion of positive tests among tests performed,
- How early HIV diagnosis occurs in the course of the disease.

**Fig. 1:**
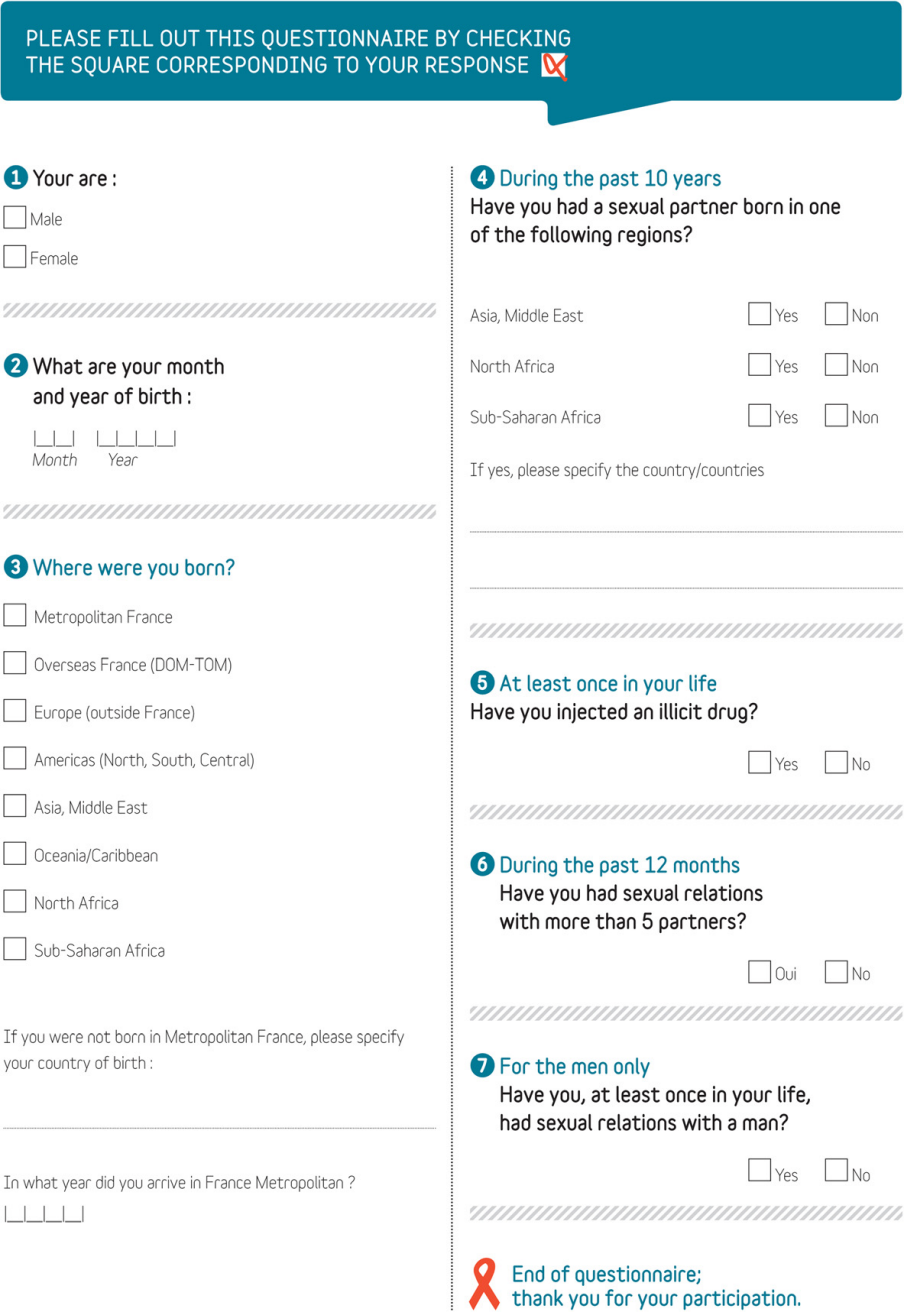
DICI-VIH questionnaire

During the period with nurse-driven targeted HIV screening, the implementation of nurse-driven targeted HIV screening is described by the proposition rate and completion rate of the DICI-VIH questionnaire (Fig. 1), the rate of patients found to be at risk, the test offering rate, the acceptance rate, the screening rate, the rate of positive tests and the evaluation of factors associated with patient refusal to be tested. During this period, the acceptability of nurse-driven targeted HIV screening by providers and patient perceptions of the process are assessed.

The costs and cost-effectiveness of the 2 strategies are also compared.

## Methods

### Study design

The DICI-VIH study is a multicentre (*n* = 8), cluster-randomized, two-period crossover trial.

**Fig. 2:**
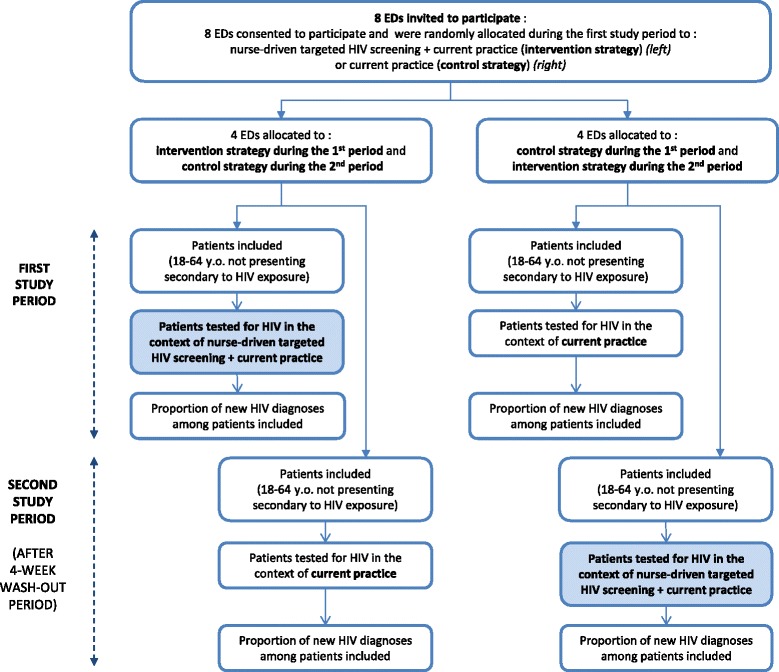
DICI-VIH flow diagram following CONSORT
- Nurse-driven targeted HIV screening combined with current practice. Nurse-driven targeted HIV screening by nurses consists in obtaining information on patient risk status from the DICI-VIH self-reported risk assessment questionnaire (Fig. 1) and, when applicable, offering, performing and delivering the result of a capillary HIV rapid test (Fig. 3). This strategy is further referred to as the intervention strategy.

The 2 strategies under comparison are:

- Current practice alone, consisting of physician-directed HIV diagnostic testing, which involves a medical interview and HIV test. This strategy is further referred to as the control strategy (Fig. 2).

**Fig. 3:**
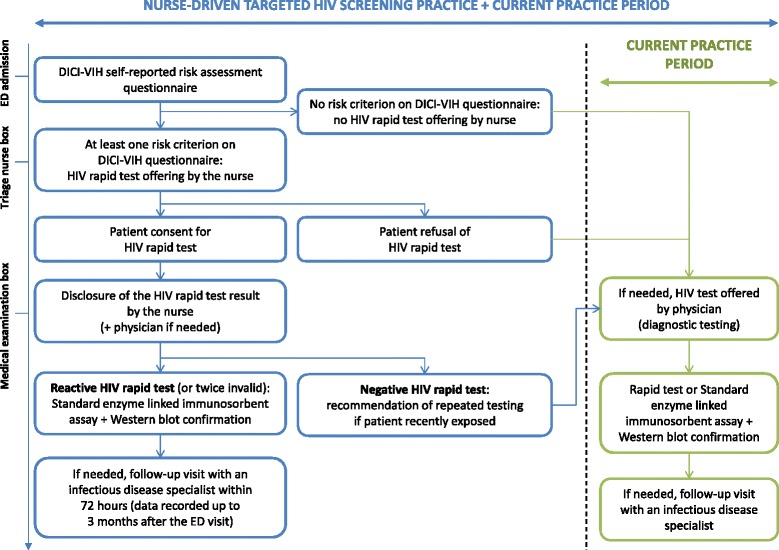
DICI-VIH flow chart

EDs were identified before randomization. Eight EDs were selected and consented to participate (see Study settings). The randomization process assigned to each ED which strategy is to be applied in the first period (the alternative strategy being applied in the second period), with half of the clusters applying the control strategy in the first period, and the remaining half applying the intervention strategy (Fig. 2). The allocation schedule was computer generated. Both study periods are separated by a 4-week washout period. An equal number of participants had to be included in each centre for each period; thus the duration of the study periods vary per ED and per period.

### Study outcomes

#### Primary outcome

The main outcome measure is the proportion of new HIV diagnoses among 18 to 64 year old patients presenting to the EDs (with the exception of those presenting secondary to HIV exposure) during the inclusion periods.

#### Secondary outcomes

The secondary outcomes are as follows:

- Proportion of patients newly diagnosed HIV positive who present for a specialist consultation within the 3 months following the test among those newly diagnosed HIV positive,
- Proportion of new HIV diagnoses among tests performed,
- How early diagnosis occurs in the course of the disease, defined as the proportion of patients that are newly diagnosed with CD4 counts >200/mm^3^ with no HIV-related symptoms, CD4 counts >350/mm^3^ with no HIV-related symptoms, and CD4 counts >500/mm^3^ with no HIV-related symptoms.

During the intervention period, the implementation of nurse-driven targeted HIV screening strategy is described by:

- The distribution rate of the DICI-VIH questionnaire: number of distributed questionnaires among included patients unaware of their HIV status and whose clinical presentation is compatible with the completion of the questionnaire,
- The completion rate of the DICI-VIH questionnaire: number of questionnaires completed among included patients unaware of their HIV status and whose clinical presentation is compatible with the completion of the questionnaire,
- The rate of patients found to be at risk: number of patients found to be at risk among included patients who filled the questionnaire,
- The test offering rate: number of patients who were offered a rapid test by nurses among patients found to be at risk,
- The acceptance rate: number of patients who accepted to be tested among patients who were offered a rapid test,
- The screening rate: number of rapid tests performed by nurses among patients found to be at risk,
- The rate of positive tests and rate of confirmed positive tests among rapid tests performed by nurses,
- The factors associated with patient refusal to be tested.

During this period, the acceptability of nurse-driven targeted HIV screening by providers and patient perceptions of the process are described qualitatively, as detailed in section 9.

The costs and cost-effectiveness of each strategy are also evaluated and are detailed in section 9.

### Study settings

The evaluation of nurse-driven targeted HIV screening is conducted in the metropolitan Paris area *(Ile-de-France)*, which is one of the most affected regions of France, with 42 % of France’s new HIV diagnoses annually [1, 9]. The selection of public hospital EDs was undertaken based on the overall high risk of exposure to HIV of their respective patient populations. Using the data from a previous study undertaken in 29 EDs reflecting the diversity of the ED settings in the Paris metropolitan area [15], 8 centres were selected as 8 % of their patients met the main targeted screening criteria (MSM, Sub Saharan African origin). The heads of the 8 EDs and their nurse supervisors were contacted and asked to participate by the research team. They all agreed to participate.

### Study participants

All patients aged 18 to 64 consulting in the ED during the recruitment periods are included, with the exception of those presenting secondary to HIV exposure through sexual or blood contact of less than 48 h.

During the nurse-driven targeted HIV screening period, the DICI-VIH questionnaire is distributed to patients unaware of their HIV status and whose clinical presentation is compatible with providing consent and with the completion of the questionnaire (the questionnaire is not distributed to patients with acute life-threatening conditions, altered consciousness, severe neuropsychiatric disorders, language barriers or who are under arrest).

### Study strategies

#### Control strategy

During the control period, the physician can offer to perform an HIV test (rapid test or standard enzyme linked immunosorbent assay and Western blot confirmation) based on the presence of HIV-related symptoms, following current practice (Fig. 3).

#### Intervention strategy

During the intervention period, the HIV rapid test is offered to patients identified as being at risk of HIV exposure based on their answers to the DICI-VIH questionnaire (at least 1 YES to the 5 following questions: lifetime exposure to male-to-male sexual contact, >5 sexual partners in the last 12 months, Sub-Saharan African origin or partner from a Sub-Saharan country in the last 10 years, lifetime injection drug use), Fig. 1. The triage nurse offers screening prior to the medical examination (Fig. 3).

Once the patient provides consent, the rapid test (VIKIA HIV1/2, bioMérieux, Marcy l’Etoile, France) is performed. If the result is reactive or if 2 sequential rapid test results are invalid, the nurse performs a blood draw for standard enzyme linked immunosorbent assay and Western blot confirmation. A follow-up visit with an infectious disease specialist (who has agreed to serve as a referral in the study) is arranged within 72 h. The infectious disease specialist can contact the patient by phone if he/she does not attend the initial appointment.

If the rapid test is negative, the nurse recommends repeating the test when exposure is recent.

If the triage nurse does not offer the test to a patient because he/she does not meet risk criteria or declines the test or if the triage nurse omits to offer it, the physician can offer an HIV test to patients presenting with HIV-related symptoms, following current practice.

#### Study procedures

Prior to the start of the intervention period with nurse-driven targeted screening, staff members (nurses, nurse supervisors, physicians, nurse assistants) of the corresponding ED will have participated in a 60-min training session organized by the research coordinator and an infectious disease specialist. The training session includes an educational lecture, an HIV rapid test demonstration, hands-on practice, and information about how to disclose test results, whether negative or reactive.

In each participating ED, the nurses are supported by a clinical research nurse or clinical research assistant and are responsible for daily patient inclusion and for collecting study data in the electronic case-report form (eCRF).

The research coordinator meets the ED teams bi-monthly to discuss any issues related to the protocol and to evaluate study progress. In addition, a one-page study newsletter is regularly given to the ED teams.

#### Designing the DICI-VIH self-reported risk assessment questionnaire

The DICI-VIH self-reported risk assessment questionnaire (Fig. 1), adapted to the French population, was designed in 2012 by an expert panel to help identify patients who have an increased probability of undiagnosed HIV infection. The questionnaire is based on variables associated with HIV infection (male-to-male sexual contact, multiple partners, country of birth and partner’s country of birth, lifetime injection drug use) [35, 41, 42]. Question wording was similar to that used for the Denver score [31, 43] and in pre blood donation assessments (France, United States, United Kingdom) so as to ensure the questionnaire’s acceptability.

To our knowledge, no score has been developed in France to predict the likelihood of unknown positive HIV status in the general population. Previous studies on non-targeted screening in metropolitan Paris failed to provide sufficient information to enable the building of such a score due to the small number of identified positive HIV cases [15, 20]. Furthermore, the scores and predictive factors published in the international literature cannot be used as they were most often developed in the United States and are not applicable to the French context [32, 43–51].

The DICI-VIH questionnaire was tested in February 2013 in one study centre. Participants completed the 7-item DICI-VIH questionnaire and a 6-item survey assessing its acceptability on a 4 point-scale rating their understanding of- and comfort with the questions. This questionnaire was submitted to patients presenting to the ED during 3 half days in the same week. It was completed by 52 of the 54 patients (24 male, 30 female) who were invited to participate, with a response rate of 96 %. Only 2 patients refused to participate due to their clinical presentation, which was not compatible with the completion of the questionnaire. No difficulties in answering the questionnaire or issues of confidentiality were identified. Ninety-eight per cent of the participants (51/52) were comfortable with the questions and were willing to disclose their HIV risks knowing that the healthcare staff would see the information. Only 1 patient declared that he was not comfortable answering the question relating to male-to-male sexual contact. 18 % of patients disclosed at least 1 risk (*n* = 2 for male-to-male sexual contact, *n* = 4 for > 5 sexual partners, *n* = 1 for Sub Saharan African origin, *n* = 1 for partner from a Sub Saharan country, *n* = 1 for injection drug use).

### Data collection and management

During both periods, general data of patient flow are collected on a daily basis in each centre by the research team on a paper CRF, including:

- Total number of patients,
- Number of patients aged 18 to 64 not presenting secondary to HIV exposure,
- Number of patients aged 18 to 64 not presenting secondary to HIV exposure who are tested for HIV (either rapid test or Standard enzyme linked immunosorbent assay) as ordered by a physician,
- Number of patients whose HIV diagnosis is confirmed to be positive by serology,
- Number of patients newly diagnosed HIV positive presenting for a specialist consultation and their characteristics (CD4 counts, viral load, HIV, HBV, HCV and Syphilis serological status and their clinical presentation). These data are downloaded with the help of the laboratory personnel in each hospital and the infectious disease specialist.

Additionally, during the nurse-driven targeted HIV period, data are also recorded by the research team on a paper CRF:

- Number of patients aged 18 to 64 not presenting secondary to HIV exposure, unaware of their HIV status and whose clinical presentation is compatible with the completion of the questionnaire,
- Number of patients who were distributed the DICI-VIH questionnaire and who filled it out,
- Number of patients found to be at risk,
- Number of patients found to be at risk and who were offered a rapid test by a nurse,
- Number of patients found to be at risk and tested by a nurse,
- Number of patients tested with a reactive rapid test (confirmed or not confirmed positive by serology),
- Number of patients whose HIV diagnosis is confirmed to be positive by serology.

With the help of the research team, nurses collect individual data from the DICI-VIH questionnaire (patient risk assessment on the patient page (Fig. 1) and offering rate, acceptance rate and screening rate on the nurse page) on an e-CRF. The quality of these data is controlled on site by the monitoring team.

Upon study completion, ED flow data, including patient baseline characteristics, degree of severity and time spent in the ED will be extracted from the electronic patient-level ED database.

A data manager collects all study data on a secure password-protected server. A quality team, independent from the coordination team, will perform an internal audit of data completeness and will ensure the individual data matches the data collected on the e-CRF.

### Sample size

We assumed that the crossover design, which results in matched-pair data within each centre (increased statistical power of the comparison test), and the effect of the cluster study design (increased between-cluster variability decreasing the statistical power) cancel each other out, see [52] webappendix p1, [53].

The sample size calculation was performed with a comparison between 2 proportions with the hypothesis of superiority.

We hypothesize that, during the intervention period, the proportion of newly diagnosed HIV patients is similar to that found in non-targeted screening combined with current practice. Based on previous data [15], the proportion of new HIV diagnoses was expected at 1.04 and 3.38 per 10,000 patients, in the control strategy and in the intervention strategy respectively. Accordingly, a study sample of 140,000 patients which corresponds to the screening of 8,750 patients per centre and per period, would lead to a statistical power of 80 % with a 5 % two-sided type 1 error rate, using Fisher’s exact test (Pass v11.0.1 software [54]).

### Statistical analysis

The statistical analysis will follow the intention-to-treat approach. A statistical analysis report will be written to describe all the findings according to the CONSORT statement recommendations [55].

The baseline characteristics of centres and patients will be described for each intervention group. Categorical variables will be described as numbers and percentages. Continuous variables will be reported using means and standard deviations or medians and interquartile ranges.

For the primary outcome, we will use generalized linear mixed modelling (Poisson mixed model) to provide statistical estimates controlling for each cluster, with the strategy intervention as a fixed effect and clusters as a random effect. The impact of the implementation order of intervention and control organization in the 2 periods on this outcome will be assessed. Additional sensitivity analysis could be performed with a permutation test.

Given the low rate of false positive rapid tests, any missing value (reactive rapid test not confirmed) will be considered as a success (positive test) in the analysis. Additional sensitivity analyses will also consider missing values as 1) success in the intervention group (HIV+ diagnosis confirmed) and failure in the control group (HIV- diagnosis confirmed); 2) failure in the intervention group and success in the control group.

The secondary outcomes regarding the presentation of patients newly diagnosed HIV positive for specialist consultation within 3 months and the rate of positive tests will be compared in the 2 groups using Pearson’s χ2 test or Fisher’s exact test when applicable. Any missing value for the rate of positive tests (reactive rapid test not confirmed) will be considered as a success (positive test).

Early diagnosis measured as the proportion of patients with CD4 counts >200/mm^3^ with no HIV-related symptoms will be compared between the 2 groups using Pearson’s χ2 test or Fisher’s exact test when applicable. The 2 other thresholds (>350/mm^3^, >500/mm^3^) with no HIV-related symptoms will also be explored. The missing values will not be replaced.

Results of the DICI-VIH questionnaires will be described.

### Additional analyses

#### Factors associated with patient refusal of nurse-driven targeted HIV screening

In half the centres, all patients who have completed the DICI-VIH questionnaire, are at risk of HIV exposure and eligible for nurse-driven targeted HIV screening are considered over randomly selected 32-h observation periods of ED activity during 7 consecutive days. The patient characteristics reported in the DICI-VIH questionnaire, history of HIV testing and perceived HIV risk will be compared between patients refusing to be tested and patients accepting the rapid test in the context of nurse-driven targeted HIV screening. A descriptive analysis of these data will be performed.

#### Patient perceptions of nurse-driven targeted HIV screening

The respondents’ perceptions of the DICI-VIH questionnaire, of being offered an HIV rapid test, of result disclosure and of HIV screening in general are collected over a week-long period in 4 of the centres. The study team interviews all the patients who fill out the DICI-VIH questionnaire using a face-to-face questionnaire. A descriptive analysis of these data will be performed. The patients refusing to fill the DICI-VIH questionnaire are not considered in this study; this is a limitation of the evaluation.

#### Acceptability of nurse-driven targeted HIV screening by providers

The acceptability of nurse-driven targeted HIV screening by providers will be studied using an ethnographic approach (direct observation, in depth interviews and questionnaires). The objectives of this qualitative section are to 1) identify individual, team-related and structural factors that might influence the implementation of the intervention, 2) evaluate how the staff perceived the implementation of the strategy (during the trial and in routine practice) and 3) evaluate the nurse’s role and involvement in the process.

In each centre, nurses answer a short questionnaire before and after the study completion in order to evaluate their perceptions of the intervention strategy and if/how their perceptions evolve after the study. The questions refer to nurse competences related to the intervention strategy and to the potential long-term implementation in EDs. In each centre, at the end of the study, individual in depth interviews with nurses, nurse assistants, nurse supervisors, physicians and directors of nursing practice are conducted in order to qualitatively collect their perceptions after the implementation of the evaluated strategy.

With the help of the questionnaires, in depth interviews and direct observation during the trial conducted by the research coordinator, factors influencing the participation in the nurse-driven targeted HIV screening are collected. These factors will be analysed in association with the centre's overall nurse test offering rate.

#### Cost and cost-effectiveness evaluation

The economic evaluation will have 3 phases: 1) estimation of the intervention strategy costs per patient tested through micro costing, 2) comparison of diagnostic costs with and without targeted screening, 3) estimation of an incremental cost-effectiveness ratio if the strategy with the greatest effectiveness also has the highest cost. In this case, the effectiveness of this strategy, compared to the alternative strategy, will be expressed in terms of extra HIV patient newly diagnosed. If the intervention strategy shows both effectiveness and cost-effectiveness, we will consider modelling the impact on the epidemic's dynamics in Paris metropolitan area.

### Organization of the trial

The study adheres to the Standard Protocol Items: Recommendations for Interventional Trials (SPIRIT) statement [56].

#### Ethical aspects

The study was approved by the *Ile-de-France* XI Committee for Patient Protection (No. 13084, January 21, 2014, N°IDRCB: 2013-AO1569-36) and by the French Data Protection Authorities responsible for database security (*Comité Consultatif sur le Traitement de l’Information en matière de Recherche dans le domaine de la Santé (CCTIRS) and Commission Nationale de l’Informatique et des Libertés (CNIL)*).

#### Funding/support

The DICI-VIH study is funded by the *Agence Nationale de Recherche sur le Sida et les Hépatites Virales (ANRS France Recherche Nord&sud Sida-hiv Hépatites),* Paris, France*.* This work is supported by a grant from *Assistance Publique – Hôpitaux de Paris (AP-HP)*, “Doctorat en recherche infirmière” programme, Paris, France. Rapid tests are provided free of charge by bioMérieux, Marcy l’Etoile, France. These 3 supporting entities have no involvement in the study design, data collection, analysis, interpretation, decision to publish, or preparation of the manuscript.

#### Data monitoring

A data monitoring committee is established and brings together the contributors of the protocol, 2 external public health specialists and an ED nurse supervisor. This committee is involved in the organization of the trial and reviews the quality of the data collected. During the trial, the members can decide which measures to adopt in case of unforeseen circumstances.

## Discussion

In countries with concentrated epidemics, targeted HIV screening strategies have not been sufficiently evaluated [31]. The DICI-VIH trial was designed to assess the effectiveness and cost-effectiveness of nurse-driven targeted HIV screening compared to routine practice in French EDs. To date, no multicentre randomized controlled trial has evaluated the effectiveness of targeted HIV screening compared to current practice in health care settings.

Several features of the trial are of particular interest. First, the trial will evaluate the feasibility of a targeting tool used by nurses on a large panel of patients in the context of EDs. Following the example of a previous study, which used a self-administered questionnaire [15], the present study is based on the DICI-VIH self-reported risk assessment questionnaire, thus avoiding having to verbally ask patients personal and sensitive questions. Moreover, in order to minimize the impact of the screening strategy on the clinical care process, the questionnaire is distributed during patient admission and the triage nurses receive patients who have already filled out the DICI-VIH questionnaire.

Secondly, this study enables the evaluation of the intervention’s effectiveness as well as the staff’s perceptions on the acceptability of the intervention by using a mixed method approach (quantitative and qualitative data). Indeed, both patient and staff perceptions of the intervention are necessary prerequisites for the successful implementation of a new HIV screening program [57].

Thirdly, in the context of limited financial resources, it is essential to evaluate public health interventions before their implementation. The costs and cost-effectiveness of nurse-driven targeted HIV screening will therefore be evaluated.

Fourth, the chosen cluster-randomized and crossover design has a methodological advantage. It reduces the risk of contamination between both strategies. With individual randomization, nurses would intermittently apply the targeted strategy and the risk of contamination may decrease observed outcome differences between the 2 strategies. Moreover, this discontinuous organization could never be applied in routine practice in EDs, raising major concerns in terms of generalizability of the results of such a study.

There are some limitations to consider with the present study design. First, due to the consecutive inclusion periods in this cross-over study, there could be a risk of inter-period contamination in the centres that received the intervention first. The risk will be assessed during the analysis. However, this risk should be low as the periods are separated by a 4-week washout period and because providers are different in the 2 groups. Indeed, screening is offered by nurses during the intervention period whereas only physicians are involved during the control period. Second, in a trial requiring active patient and provider participation, the implementation of the 2 strategies will be associated with some variability between clusters regarding the proportion of new HIV diagnosis. This is not a default on the contrary; the cluster-randomized two-period crossover design is actually an asset for the estimation of between-cluster variability [58]. Moreover, assessing and understanding differences in intervention effectiveness and adherence from one ED to another is a core part of the analysis. In addition, the factors associated with screening refusal will be evaluated in the present study. They have already been explored in studies on non-targeted screening and results suggest that refusal is not associated with belonging to the highest risk groups [15, 59–61]. Third, the number of clusters (*n* = 8) is relatively small, with an associated limited statistical power of the test of interaction between intervention order and intervention effectiveness [62].

If the effectiveness of the intervention strategy is confirmed, the results of this trial could contribute to the development of recommendations and nurse-driven targeted HIV screening could be generalized in the French EDs of areas with high HIV prevalence. Finally, although the results may not be directly applicable to other countries, they could be helpful for decision makers in countries with concentrated epidemics.

## Conclusion

To our knowledge, the DICI-VIH study is the first large randomized controlled trial designed to assess the benefits of nurse-driven targeted HIV screening in EDs. Findings from this study will contribute to the development of HIV screening strategies, and to the promotion of nurse participation in prevention and public health programs.

### Trial status

Inclusions started in June 2014. Initial results are expected by mid-2016.

## Abbreviations

CRF: Case Report Form
DICI-VIH: *Dépistage Infirmier CIblé du VIH,* Nurse-driven HIV targeted screening
eCRF: Electronic Case Report Form
MSM: Men who have Sex with Men
